# Prevalence and factors associated with hypertension in Burkina Faso: a countrywide cross-sectional study

**DOI:** 10.1186/s12889-016-3926-8

**Published:** 2017-01-11

**Authors:** Joseph Kouesyandé Soubeiga, Tieba Millogo, Brice W. Bicaba, Boukare Doulougou, Séni Kouanda

**Affiliations:** 1 Institut Africain de Santé publique (IASP) & Ministry of Health, Ouagadougou, Burkina Faso; 2 Institut Africain de Santé publique (IASP) & Institut de recherche en sciences de la santé (IRSS), 03 BP 7102 Ouagadougou, Burkina Faso; 3 Institut de recherche en sciences de la santé (IRSS), Ouagadougou, Burkina Faso; 4 Institut Africain de Santé publique (IASP), USTA, Saaba, Bâtiment C, 12 BP 199 Ouagadougou, Burkina Faso

**Keywords:** Hypertension, Prevalence, Risk Factors, Burkina Faso, NCDs

## Abstract

**Background:**

High blood pressure (HBP) is an increasing public health issue for developing countries. HBP is an important contributing factor to many non-communicable diseases that were until very recently thought to be rare in developing countries. There is not enough evidence on its burden and risk factors in Africa. We report in this study on the prevalence and factors associated with HBP in the adult and active population of Burkina Faso from a nationally representative sample.

**Methods:**

We conducted a secondary analysis of data from the World Health Organization (WHO) Stepwise approach to Surveillance(STEPS) survey on the prevalence of major risk factors for non-communicable diseases in Burkina Faso. This survey was conducted between September 26 and November 18, 2013 and involved a nationally representative sample of 4,800 adults aged 25 to 64 years. The risk factors were identified using a binary logistic regression in STATA Version 13.1 software.

**Results:**

The analysis was conducted on a sample of 4629 participants of whom 72.18% lived in rural areas. The overall prevalence of hypertension in Burkina Faso was 18% (95% CI: 16.19%–19.96%). In urban areas the prevalence was 24.81% (95% CI 20.21%–30.07%) and 15.37% (95% CI 13.67%–17.24%) in rural areas. Increased Body Mass Index (BMI) and older age were consistently associated with higher odds of HBP in both residential areas. In addition, being of male sex, fat intake, family history of HBP and low level of HDL cholesterol were significantly associated with increased odds of HBP in rural residents.

**Conclusion:**

The prevalence of hypertension is high in Burkina Faso with roughly one person in five affected. There is a predominant burden in urban areas with prevalence of ten-point percent higher compared to rural area. Modifiable risk factors should be targeted with appropriate and effective strategies to curb the rising burden of hypertension and its consequences.

## Background

High blood pressure (HBP) is a major global public health issue according to WHO [1, 2] and its prevalence varies importantly across settings [3]. In 2008, according to WHO, the global prevalence of hypertension in adults aged 25 years and more was around 40% and was higher in the African Region (46%) [1].

In Africa, the prevalence of hypertension varies from one country to another and across residential areas [4]. A prevalence of 16.9% in the adult population aged 15 and over was reported in Ethiopia [5] against 36.7% in Ghana [6]. In urban areas the prevalence was 23% in Benin [7] against 54.6% in Ghana [8]. In rural areas, the prevalence was 14.6% in Uganda [9] and 44.5% in Nigeria [10]. The risk factors for HBP are well known. However, there are important setting’ specificities in their importance [4, 7, 9, 11–26].

Well documented risk factors for HBP comprise non-modifiable factors such as age, sex and family history of HBP; behavioral risk factors such as salt intake, alcohol consumption, smoking, physical inactivity; socio-demographic factors such as the place of residence, educational level, economic status, marital status; and metabolic factors such as diabetes, obesity, dyslipidemia. The context specificity must be accounted for and each country should identify risk factors that are overriding in its context to better guide public health policies, especially countries with limited resources like Burkina Faso. Previous small size studies on the risk factors for hypertension were conducted in Burkina Faso in Ouagadougou in 2004 among adults aged 35 and more [27] and in the Kaya Health and Demographic Surveillance System (KayaHDSS) in 2012 among adults aged 18 and older [28].

Burkina Faso, like other developing countries, is facing the emergence of non-communicable diseases (NCDs) and HBP is a major contributing factor to the burden of NCDs. Its prevalence was estimated at 29.4% in Burkina Faso in 2008 by WHO [29]. To address the lack of comprehensive data on Non-Communicable Diseases (NCDs) and their risk factors a first national survey was conducted in2013 [30] using the WHO STEPwise approach to risk factors surveillance(STEPS) for NCDs. The STEPS survey is a simple, standardized method for collecting, analyzing and disseminating data in WHO member countries [31]. It covered a representative sample of the adult population of Burkina Faso. The first analyses of this survey data were mainly descriptive [30]. We report here on the prevalence of hypertension and the associated factors using a secondary analysis of the STEPS survey data.

## Methods

### Study settings

Our research was conducted in Burkina Faso, a landlocked country in West Africa. With a total land area of 272 967.47 km^2^, the country is limited in the North and West by Mali, in the East by Niger and in the South by Benin, Togo, Ghana and Côte d’Ivoire. Administratively, the country is divided into 13 regions, 45 provinces, 351 municipalities (49 urban municipalities and 302 rural municipalities) and 8228 villages. In 2013, the population was estimated at 17,271,583 inhabitants. The majority of the population (77.30%) lives in rural areas on agriculture and livestock [32].

The population is predominantly young with the 0–15-year old estimated at 46.4%. The total fertility rate (TFR) is 6.0 (2010) for the entire country. Life expectancy at birth was 57 years in 2006. The country witnessed persistent high burden of diseases with a mix of endemic and epidemic diseases. Indeed, the country is regularly confronted with outbreaks such as cerebrospinal meningitis and measles [30] while NCDs constitute a raising public health problem for the health system. According to the 2014 WHO report on the epidemiological profile of NCDs, the prevalence of hypertension and obesity in Burkina Faso was estimated at 29.4 and 2.3% respectively [29].

### Study design

We carried out an analytical cross-sectional study that consisted in the secondary analysis of the baseline data of the WHO STEPS survey conducted in Burkina Faso in 2013.

### Study population

The research participants were composed of adults of both sexes aged 25 to 64 years who had been residing in the country for at least six (06) months on the day of the survey. We excluded from the research, people with disabilities hampering their ability to answer the questions (serious mental disorder, hearing or intellectual disability).

### Sampling

The sampling frame used in the STEPS survey was the enumeration areas (EAs) from the 2006 general census of the population and housing (GCPH 2006) and updated in 2010 during the demographic and health survey in Burkina Faso [32]. Enumeration areas are zones of roughly similar size in terms of number of households into which the entire population of Burkina Faso was divided by the national institute of statistics and demography for survey purposes. The sample was stratified to reflect adequate representation of urban and rural areas in the 13 administrative regions. In each stratum, a three-stage cluster sampling was performed. The first stage was the selection of EAs through a random sampling with a probability proportional to the size of the EAs per administrative region. In total, 240 EAs were selected including 186 EAs for rural areas and 54 EAs for urban areas. At the second stage, the selection of 20 households in each of the EAs selected at the first stage using a simple systematic sampling. At the third stage, the random selection of an individual aged 25–64 years in the households selected using the method of Kish [33]. The sample size was calculated using the formula N = z_α_^2^ P (1-P)/e^2^ where z = α error (5%); P = prevalence of HBP in previous findings in the country (29,4%); e = precision (5%). Given subgroup analyses of 8 groups (4 age groups and 2 sex groups or urban-rural groups), a cluster sampling design (described above) with a design effect set at 1.5, and 20% non-response, the sample size was estimated to be 4785: $n=\left(\frac{Z_{\alpha}^2*P\left(1-P\right)}{e^2}*1.5*8\right)/0.8$.

**Fig. 1:**
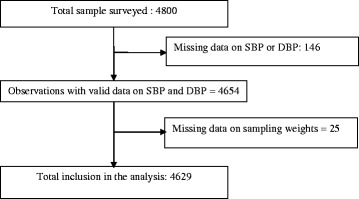
Diagram flow of the study participants. This figure depicts the criteria that were applied in selecting the study participants and the numbers that were affected by these criteria

The STEPS survey of Burkina Faso was thus conducted on a representative sample of 4800 individuals. After excluding from the database individuals with missing information on blood pressure and sampling weight, 4629 (96.43%) individuals were finally included in our analysis. Figure 1 summarizes the process of inclusion in the analyses.

Considering a 29.4% HBP prevalence in non-exposed people [29] and a 35.28% HBP prevalence in exposed ones, at the type 1 error threshold of 5%, we would have a 99% statistical power with the size of 4629 individuals.

### Data collection

The data were collected using standardized WHO STEPS questionnaires implemented in laptop computers. The information was collected in households via direct interviews in the language most spoken by the respondent. The data collected were related to socio-demographic information, behavioral measures, and measures of physical and biochemical parameters. The data collection team consisted of supervisors and interviewers. The supervisors were statisticians, epidemiologists and clinicians. The interviewers were nurses and medical students at the end of their training paths and who had proven experience in population surveys. This field staff was trained to standardize data collection. The questionnaire was pre-tested on the field before being used for the national survey.

Blood pressure was measured with an electronic blood pressure monitor (OMRON HEM-705 brand PC, Tokyo, Japan). Each interviewed individual had his blood pressure systematically measured three times by the same technician designated by the team. The systolic and diastolic blood pressures were measured at the right arm after a 15-min rest without crossing legs. The cuff was placed above the elbow between 1.2 and 2.5 cm above the elbow bend and maintained at the same level with the heart during the measurement. The measurements were separated by 5-min intervals. The mean of these three readings was considered for the definition of HBP.

### Study variables

The dependent variable in our study was the hypertension. We considered as hypertensive person any participant with systolic blood pressure (SBP) higher than or equal to 140 mmHg and/or diastolic blood pressure (DBP)higher than or equal to 90 mmHg or participants who reported current antihypertensive therapy use [28].

The independent variables included: demographics (age, sex, marital status, place of residence, occupation, education level), personal and family history of hypertension, behavioral measures (consumption of alcohol, fruit, vegetables, fat, current consumption of smoked and non-smoked tobacco products, the weekly amount of meals consumed outside the household, intensity of physical activity), physical measures (weight, height) and biochemical measures (fasting glucose, total cholesterol levels, HDL-Cholesterol).

### Data analysis and processing

Some independent variables were transformed, recoded and grouped for analytical purposes. Table 1 summarizes the different transformations that were carried out. Analyses were carried out separately for urban and rural residents.

**Table 1:** Definition of recoded exposure variables

Variables	Categories
Age groups	«25 to 34 years» «35 to 44 years» «45 to 54 years» «55 to 64 years»
Education	«No education» «Primary» «Secondary/Tertiary»
Marital status	«Single» «Married» «Divorced/widowed»
Occupation	«Wage earner» «self-employed» «Jobless» «House maker»«None» «Vegetable oil» « butter, lard or fat, margarine»
Fat intake	«None» «Vegetable oil» « butter, lard or fat, margarine»
BMI	Underweight (<18.5); normal ([18.5–25[); overweight ([25–30[); obese (≥30)
Fasting glucose	Normal (<6.1 mmol/l); High glucose (≥6.1 mmol/l)
Total cholesterol	Normal (<5.2 mmol/l); High cholesterol (≥5.2 mmol/l)
HDL cholesterol	Low (<1.03 mmol/l for men & <1.29 mmol/l for women) High (≥1.03 mmol/l for men & ≥1.29 mmol/l for women)
Physical activity	o High intensity activity: high intensity activities require an energy expenditure greater than 6 Metabolic equivalent of Task (METs). Examples include playing soccer, digging whole, regular swimming, farming etc. o Moderate intensity activity: Moderate intensity activities require an energy expenditure of approximately 3–6 METs. Examples include cleaning, vacuuming, polishing, gardening, cycling at a regular pace or horse riding etc. o Low intensity activity: refers to persons who do not qualify for the above classifications. Here are included those with limited and no physical activity.

The qualitative variables were described using proportions and we calculated the means and standard deviations for continuous variables. Age-standardized prevalence of HBP was calculated using the WHO standard population [34]. The Fisher Exact test was used in univariable analysis to test for association between outcome and categorical covariates. Unadjusted ORs were calculated for explanatory variables by using the logit function in STATA. All these analyses were carried out using the survey function (svy) with the sampling weights option in STATA to account for the sampling design. Candidates predictors selection in the multivariable analysis was based on the relevance in the literature. In multivariable analysis, a logistic regression model was fitted using stepwise backward regression modelling. Robust standard errors were computed to account for the clustered nature of the data; Successive models were compared using the Akaike’s Information Criterion (AIC) and Bayesian’s Information Criterion (BIC). The rule of parsimony [35] was applied to choose the final model whose goodness of fit was checked.

## Results

In the study sample, 3600 (72.18%) were rural residents. The population was predominantly young with the 25–34 years’ age group representing 41.98%. Women represented 54.15%. Table 2 presents the socio-demographic characteristics of the study sample stratified according to the area of residence.

**Table 2:** Socio-demographic characteristics of the study sample

Variables	Population		
	Urban n (%)	Rural n (%)	Total n (%)
Age groups			
25 to 34	460 (42.06)	1635 (41.95)	2095 (41.98)
35 to 44	276 (27.84)	888 (27.76)	1164 (27.78)
45 to 54	184 (19.18)	650 (18.57)	834 (18.74)
55 to 64	109 (10.92)	427 (11.72)	536 (11.50)
Sex			
Male	457 (42.13)	1773 (47.29)	2230 (45.85)
Female	572 (57.87)	1827 (52.71)	2399 (54.15)
Education			
None	508 (53.43)	3067 (86.55)	3575 (77.33)
Primary	278 (25.33)	438 (11.42)	716 (15.29)
Secondary/Tertiary	243 (21.24)	87 (2.03)	330 (7.38)
Marital status			
Single	171 (15.31)	156 (3.5)	327 (6.79)
Married	772 (76.32)	3218 (91.45)	3990 (87.24)
Divorced/Widowed	85 (8.37)	222 (5.05)	307 (5.97)
Occupational status			
Wage earner	192 (17.02)	66 (1.55)	258 (5.86)
Self-employed	480 (44.28)	2730 (75.09)	3210 (66.52)
Jobless	101 (9.6)	44 (0.99)	145 (3.38)
House maker	256 (29.1)	760 (22.37)	1016 (24.24)

The Table 3 presents the prevalence of HBP per age groups and per residential areas. The overall prevalence of HBP in individuals aged 25–64 years in Burkina Faso was 18% (95% CI :16.18–19.96), the prevalence was 24.81% (95% CI: 20.13 to 30.17) in urban residents and 15.37% (95% CI:13.67 to 17.24) in rural residents. This difference in prevalence between urban and rural areas was statistically significant (*p* < 0.001). The HBP prevalence varied from one administrative region to another (result not shown), it was lower in the North Central region (7.16%) and higher in the Central region (29.27%).

**Table 3:** prevalence of HBP in urban and rural adult population aged 25-64 years

	RURAL		URBAN		Total	
	n	HBP % (95%CI)	n	HBP % (95%CI)	N	HBP % (95%CI)
Age (years)						
25 to 34	1635	8.81 (7.20–10.73)	460	14.16 (10.31–19.13)	2095	10.30 (8.68–12.17)
35 to 44	888	15.76 (12.82–19.23)	276	19.77 (14.58–26.23)	1164	16.88 (14.28–19.84)
45 to 54	650	20.16 (16.42–24.51)	184	37.47 (27.60–48.51)	834	25.09 (21.04–29.62)
55 to 64	427	30.37 (25.42–35.82)	109	56.49 (42.75–69.30)	536	37.27 (31.93–42.95)
Total	3600	15.37 (13.67–17.24)	1029	24.81 (20.13–30.17)	4629	18 (16.18–19.96)
Total adjusted^a^		17.05 (15.27–18.82)		27.4 (22.49–32.30)		20.09 (18.21–21.97)

^a^Prevalence adjusted to WHO world standard population

The HBP prevalence increases along with age. This positive correlation between hypertension and age was observed in both rural and urban areas. In urban areas the prevalence increased from 14.16% for the 25–34 years ‘age group to 56.49% for the 55–65 years’ age group and in rural areas the prevalence increased from 8.81 to 30.37% for the same age groups.

Risk factors significantly associated with hypertension under multivariable analysis in rural area were: age with higher odds of HBP as age increases (*p* <0.001), the family history of hypertension [absence of family history of HBP OR = 0.62; 95% CI:0.45 to 0.83], the consumption of butter/lard or margarine as compared to none or vegetable oil [OR = 1.98; 95% CI:1.22 to 3.22], overweight [OR = 1.87; 95% CI:1.13 to 3.10], obesity [OR = 4.63; 95% CI:2.21 to 9.69], low HDL cholesterol [OR = 1.77; 95% CI:1.31 to 2.40], female sex as compared to male [OR = 0.74; 95%CI: 0.55–0.99], and the marital status where being married as compared to single was protective [OR = 0.44; 95%CI: 0.24–0.81]. Current smoking was also a risk factor for HBP in rural area, although the association was not statistically significant [OR = 1.52; 95% CI:1.00–2.32].

In urban area, the association between covariates and HBP remain in the same direction as described above in rural area. However, only BMI and age reached quite statistical significance in urban area. All the results of the multivariable analysis on risk factors for HBP are presented in Table 4.

**Table 4:** Risk factors for HBP in urban and rural adult population aged 25–64 years

	RURAL				URBAN			
	N	HBP n (%)	COR^a^ (95%CI)	AOR^b^ (95% CI)	N	HBP n (%)	COR (95%CI)	AOR (95% CI)
Total	3600	553 (17.05)^c^			1029	245 (27.4)^c^		
Age (years)								
25 to 34	1635	147 (8.81)	1	1.00	460	66 (14.16)	1.00	1.00
35 to 44	888	146 (15.76)	1.93 (1.46–2.56)***	1.75 (1.25–2.47) **	276	59 (19.77)	1.50 (0.96–2.32)	1.40 (0.84–2.32)
45 to 54	650	129 (20.16)	2.61 (1.91–3.57)***	2.79 (1.91–4.07)***	184	62 (37.47)	3.63 (2.24–5.88)***	3.03 (1.71–5.38)***
55 to 64	427	131 (30.37)	4.52 (3.31–6.16)***	4.49 (2.99–6.73)***	109	58 (56.49)	7.87 (4.58–13.52)***	7.71 (4.07–14.60)***
Education								
None	3067	470 (15.31)	1.00	1.00	508	124 (25.05)	1.00	1.00
Primary	438	61 (14.56)	0.94 (0.67–1.32)	0.82 (0.56–1.21)	278	59 (24.46)	0.96 (0.63–1.47)	1.21 (0.72–2.00)
Secondary/Tertiary	87	19 (21.74)	1.53 (0.85–2.75)	1.06 (0.54–2.07)	243	62 (24.64)	0.97 (0.64–1.49)	1.05 (0.64–1.74)
Marital Status								
Single	156	33 (22.05)	1.00	1.00	171	26 (13.53)	1.00	1.00
Married	3218	462 (14.63)	0.61 (0.36–1.00)	0.44 (0.24–0.81)**	772	191 (25.88)	2.23 (1.33–3.73)**	1.29 (0.68–2.44)
Divorced/Widow	222	57 (24.12)	1.52 (0.62–2.03)	0.73 (0.34–1.54)	85	27 (35.17)	3.46 (1.67–7.19)	1.27 (0.47–3.39)
Sex								
Male	1773	314 (17.62)	1.00	1.00	457	119 (26.60)	1.00	1.00
Female	1827	239 (13.35)	0.72 (0.58–0.89)**	0.74 (0.55–1.00)*	457	126 (23.52)	0.86 (0.60–1.20)	0.81 (0.53–1.23)
Family history of HBP								
Yes	482	117 (23.57)	1.00	1.00	339	111 (32.70)	1.00	1.00
No	482	358 (13.97)	0.53 (0.40–0.70)***	0.62 (0.45–0.83)**	622	121 (20.85)	0.54 (0.37–0.64)**	0.67 (0.44–1.02)
Current smoking								
Yes	447	56 (12.56)	1.00	1.00				
No	3152	497 (15.74)	1.3 (0.93–1.82)	1.52 (1.00–2.32)				
Physical activity								
Low	439	95 (19.35)	1.00	1.00	237	71 (30.56)	1.00	1.00
Moderate	761	125 (17.26)	0.86 (0.62–1.22)	1.01 (0.68–1.51)	331	68 (21.57)	0.62 (0.39–0.99)*	0.77 (0.45–1.30)
High(intense)	1890	269 (14.31)	0.62 (0.52–0.93)*	0.84 (0.59–1.21)	367	78 (22.92)	0.67 (0.43–1.06)	0.93 (0.55–1.58)
Main type of fat used								
None	397	50 (11.32)	1.00	1.00				
Vegetable oil	2025	308 (14.76)	1.36 (0.93–1.98)	1.42 (0.89–2.27)				
Butter/lard/margarine	1090	179 (17.83)	1.70 (1.15–2.52)**	1.98 (1.22–3.22)**				
BMI (kg/m2)								
Underweight	434	61 (14.35)	1	1.00	66	9 (14.18)	1.00	1.00
Normal	2 637	374 (14.23)	0.98 (0.70–1.38)	1.02 (0.67–1.54)	547	105 (19.7)	1.48 (0.63–3.48)	2.0 (0.77–5.18)
Overweight	2 637	88 (24.47)	1.93 (1.26–2.95)**	1.87 (1.13–3.10)*	252	65 (30.53)	2.66 (1.10–6.42)*	3.76 (1.41–10.06)**
Obese	66	26 (42.36)	4.38 (2.34–8.19)***	4.63 (2.21–9.69)***	129	63 (48.68)	5.74 (2.31–14.26)***	8.01 (2.94–21.84)***
HDL cholesterol								
High	2782	389 (13.71)	1.00	1.00	715	163 (24.29)	1.00	1.00
Low	2782	163 (21.20)	1.70 (1.32–2.16)***	1.77 (1.31–2.40)***	292	77 (26.40)	1.12 (0.77–1.62)	1.26 (0.81–1.94)

^a^ Crude Odds Ratios

^b^ Adjusted Odds Ratios

^c^ Prevalence adjusted to WHO world standard population

## Discussion

The overall prevalence of hypertension in our study was 18%. STEPS surveys conducted in several countries in the West African sub-region in similar age groups reported similar or higher prevalence of hypertension: 21.2% in Niger [36], 24.2% in Gambia [20], 26.6% in Togo [37], 27.5% in Benin [38], 34.8% in Nigeria [39], and 36.7% in Ghana [21]. The prevalence in Mali [40] was 15.9%, that is lower than the prevalence found in our study. The prevalence of hypertension in Burkina seems to be among the lowest in the West African sub-region. The prevalence of hypertension in the land-locked countries (Burkina Faso, Mali) also appears to be lower than that of coastal countries of the same sub-region. A possible explanation for this relative low prevalence is the difference in dietary habits between land-locked and coastal countries. Previous studies [27, 41] in Ouagadougou in 2003 and 2007 reported HBP prevalence of 23% in 18 years and older and 40% in 35 years and older. Another study [42] in 2011 in Ouagadougou reported a prevalence of 29.6%. The figures in these studies are higher compared to our study. We report here the results from a population-based representative sample as opposed to previous studies that were carried out on smaller samples. The prevalence of hypertension is higher in urban than rural areas with a statistically significant difference in our data. There seems to be consistent finding that the burden of hypertension remains heavy in urban settings compared to rural ones, despite recent increase in rural residents.

The predominance of hypertension in the Centre and Hauts Basins Regions may be due to the rapid and important urbanization in these two regions that host the most important cities of the country (Ouagadougou, Bobo Dioulasso). Indeed, urbanization causes changes in lifestyle (sedentary lifestyle, consumption of industrial food ready for consumption) and exposure to several risk factors in towns [7, 27]. The relative low prevalence in rural areas may be in contrary due to persistent traditional lifestyle and eating patterns [28] and lesser exposure to risk factors for hypertension. This predominance of hypertension in urban centers as compared to rural settings was reported by several studies in Africa [3, 4, 22].

Our research revealed that age and family history were associated with hypertension in urban and rural areas.

Aging reduces the elasticity of blood vessels leading to an increase in blood pressure [43]. Another possible reason is that older people pay less attention to their health or lack financial means for health care [44]. Also, the accumulation of hypertension risk factors increases along with the age of individuals [43].

Previous studies showed that HBP is a common, complex and polygenic disease whose phenotype is the result of multiple interactions between genes and the environment [45]. In our context, the environmental aspects linked to family dietary habits or cultural aspects could also explain the association between family history and hypertension. Previous studies in Burkina Faso [28] and several studies in Africa [8, 9, 13, 14] highlighted the existence of an association between hypertension and family history of hypertension.

Marital status was associated with hypertension. The single status may expose individuals to more stress and low socialization while marriage could lead to more security and stability for spouses, with less exposure to stress. Moreover, unmarried individuals may have less control over their diet as they tend to eat meals more often outside the household.

Such meals are usually saltier, fattier and contain more spices and broths [8]. Studies have shown that reducing daily salt intake lead to a decrease in blood pressure [46, 47]. An earlier study in the North Central Region in Burkina Faso showed that in rural areas marriage was a protective factor against the occurrence of hypertension [28]. In South Africa, studies also showed a relationship between marital status and hypertension [13, 14].

The consumption of fat was associated with hypertension in rural areas. The consumption of saturated fats (butter, meat, lard, fat or margarine, whole milk, etc.) is common in rural areas. Animal fat especially from pork and denatured oils from fries are reused for the cooking of food in households. A study in rural Malawi, Tanzania and Rwanda [22] reported similar findings about fat consumption.

Metabolic risk factors such as BMI (overweight, obesity), dyslipidemia (high cholesterol and low HDL-cholesterol) also showed association with HBP as it was previously reported in Burkina Faso [28] and Africa [4, 22]. The association between overweight, obesity and hypertension is well known and reducing BMI is part of the advice provided in the treatment of hypertension [43]. Increasing BMI is often associated with metabolic and endocrine disorders, which increase the risk of occurrence of hypertension [48].

We present here the results of the first national survey on the prevalence and risk factors for hypertension in Burkina Faso. The study is however entitled to some limitations. The measurement of most exposures was based on the recall of respondents and some well-known risk factors for hypertension were not included in the study because data on these variables have not been collected during the STEPS survey. Part of such variables are: average daily salt intake [46, 47]; socioeconomic status [4, 13] (poverty, social relations between individuals in the community, living conditions of households); the ethnic origin [13] which has a connection with cooking habits, psychological aspects [13, 28] (stress, insomnia, anxiety, depression), presence of other chronic diseases [28].

## Conclusion

We report on the burden of HBP and associated risk factors in a nationally representative sample of adults in Burkina Faso. Our data confirm an important burden of HBP in the entire population with higher burden for urban settings. Obesity and dietary habits are significant modifiable risk factors. Zone specific interventions are needed given the higher burden in urban centers. Health policies in Burkina Faso must henceforth account for the control of HBP.

## Abbreviations

AOR: Adjusted odds ratios
BMI: Body mass Index
BP: Blood pressure
CI: Confidence interval
COR: Crude odds ratios
DBP: Diastolic blood pressure
EA: Enumeration area
HBP: High blood pressure
HDL: High density lipoprotein
LDL: Low density lipoprotein
MmHg: mmHg millimeter mercury
mmol/l: mmol/l millimole per liter
NCDs: Non-communicable diseases
OR: Odds ratio
PDA: Personnel digital assistant
SBP: Systolic blood pressure
SPSS: Statistical package for the social sciences
WHO: WHO World Health Organization
